# Diversity and abundance of microbial eukaryotes in stream sediments from Svalbard

**DOI:** 10.1007/s00300-017-2106-3

**Published:** 2017-03-31

**Authors:** R. S. Hindshaw, M. R. Lindsay, E. S. Boyd

**Affiliations:** 1 Department of Earth Sciences, University of Cambridge, Cambridge, UK; 2 Department of Microbiology and Immunology, Montana State University, Bozeman, MT USA; 3 NASA Astrobiology Institute, Mountain View, CA USA

**Keywords:** Eukarya, Svalbard, Sediment, Glacier, Chemotrophy, Permafrost

## Abstract

Microbial eukaryotes are increasingly being recognised for their role in global biogeochemical cycles, yet very few studies have focussed on their distribution in high-latitude stream sediments, an important habitat which influences stream water nutrient chemistry. In this study, we present the first comparison of microbial eukaryotes from two different polar habitats by determining the abundance and taxonomic affiliation of 18S rRNA gene fragments recovered from four sediment samples in Svalbard: two from a glaciated catchment and two from an unglaciated permafrost-dominated catchment. Whilst there was no difference between the two catchments in terms of Rao’s phylogenetic diversity (0.18±0.04, 1SD), the glaciated catchment samples had slightly higher richness (138–139) than the unglaciated catchment samples (67–106). At the phylum level, Ciliophora had the highest relative abundance in the samples from the glaciated catchment (32–63%), but only comprised 0–17% of the unglaciated catchment samples. Bacillariophyta was the most abundant phylum in one of the samples from the unglaciated catchment (43%) but phototrophic microbial eukaryotes only formed a minor component of the glaciated catchment samples (<2%), suggesting that in these environments the microbial eukaryotes are predominantly heterotrophic (chemotrophic). This is in contrast to previously published data from Robertson Glacier, Canada where the relative abundance of chlorophyta (phototrophs) in three samples was 48–57%. The contrast may be due to differences in glacial hydrology and/or geology, highlighting the variation in microbial eukaryote communities between nominally similar environments.

**Electronic supplementary material:**

The online version of this article (doi:10.1007/s00300-017-2106-3) contains supplementary material, which is available to authorized users.

## Introduction

Northern latitude Polar environments have been shown to contain active and diverse microbial communities (e.g. Anesio and Laybourn-Parry 2012; Hamilton et al. 2013; Jansson and Taş 2014) whose compositions and associated activities are susceptible to external physical and chemical change, for example, permafrost thaw (e.g. Mackelprang et al. 2011; Liebner et al. 2015). In addition, these microbial communities have been shown to play key roles in biogeochemical cycles, in particular the carbon cycle (McCalley et al. 2014). For example, whether Arctic streams are net sources or sinks of carbon dioxide depends on the balance between autotrophic and heterotrophic organisms which also directly impacts on stream dissolved organic carbon (DOC) dynamics (e.g. Battin et al. 2008).

The number of studies that use gene or transcript sequencing to characterise microbial communities and their role in biogeochemical cycling in high-latitude systems has grown tremendously over the past decade (Anesio and Laybourn-Parry 2012; Boetius et al. 2015). Collectively, these studies have shown the presence of diverse bacterial and archaeal communities that directly impact the extent and nature of mineral weathering (e.g. Skidmore et al. 2005; Boyd et al. 2014; Hindshaw et al. 2016a), nitrogen cycling (e.g. Boyd et al. 2011; Mackelprang et al. 2011; Tveit et al. 2013), hydrogen cycling (Telling et al. 2015), sulphur cycling (Harrold et al. 2016) and carbon cycling (e.g. Boyd et al. 2010, 2014; Mackelprang et al. 2011; Tveit et al. 2013). However, there have been comparatively fewer studies conducted on microbial eukaryotes in cold, high-latitude ecosystems despite the potentially important role that they have in nutrient cycling in these environments. The few cold high-latitude terrestrial ecosystems where microbial eukaryotes have been investigated (Table 1) have focussed on their diversity in glacial cryoconite habitats (Christner et al. 2003; Cameron et al. 2012; Hamilton et al. 2013), freshwater cyanobacterial mats (Jungblut et al. 2012) permafrost soils (Coolen et al. 2011; Mackelprang et al. 2011; Tveit et al. 2013, 2014; Geisen et al. 2015), snow (Harding et al. 2011; Hamilton et al. 2013; Cameron et al. 2015), streams and porewaters (Luo et al. 2009; Crump et al. 2012), and subglacial environments (Hamilton et al. 2013). One of the most abundant phyla found in high-latitude environments are Ciliophora (Tveit et al. 2013; Geisen et al. 2015) and their presence may control the abundance of bacteria and recycling of soil organic matter (Coolen et al. 2011). In addition to recycling organic matter, Ciliophora, and heterotrophic eukarya in general, are themselves dependent on organic carbon and were found to comprise a significant fraction of active eukaryotes in a subglacial sediment sample (Hamilton et al. 2013), further indicating their potential role in carbon cycling.

**Table 1:** Compilation of previous microbial eukaryote sequencing studies from terrestrial polar environments

Reference	Environment	Location	Study Accession Number	Method$^{\text {a}}$
Bachy et al. (2011)	Snow	North Pole	HQ438100–HQ438190$^{\text {b}}$ and	Sanger
			JF826314–JF826397$^{\text {b}}$	
Cameron et al. (2012)	Cryoconite	Kangerlussuaq, Greenland	GU297612–GU298216$^{\text {b}}$	Sanger
		Midre Lovenbreen, Svalbard		
		Longyearbyen, Svalbard		
		Vestfold Hills, Antarctica		
		Signy Island, Antarctica		
Cameron et al. (2015)	Snow	Thule, Greenland	PRJEB4904$^{\text {c}}$	Sanger
		Kangerlussuaq, Greenland		
Christner et al. (2003)	Cryoconite	Dry Valleys, Antarctica	AY124360–124370	Sanger
Coolen et al. (2011)	Permafrost soil	Kuparuk River, Alaska	JF829151–JF829211$^{\text {b}}$	Sanger
Crump et al. (2012)	Soil water	Toolik Lake, Alaska	SRA049830$^{\text {d}}$	Pyrotag
	Headwater stream			
	Lake inlet			
	Epilimnion			
	Hypolimnion			
Hamilton et al. (2013)	Cryoconite	Robertson glacier, Canada	SAMN01729474–SAMN01729995$^{\text {d}}$	Pyrotag
	Snow			
	Subglacial sed.			
Harding et al. (2011)	Snow	Ellesmere Island, Canada	HQ230103–HQ230240 and	Sanger
			HQ529495–HQ529499$^{\text {b}}$	
Jungblut et al. (2012)	Cyanobacteria mats	Ellesmere Island, Canada	JN207853–JN207906$^{\text {b}}$	Sanger
Luo et al. (2009)	Glacial meltwater	Austre Brøggerbreen, Svalbard	EU050744–EU050983$^{\text {b}}$	Sanger
Mackelprang et al. (2011)	Permafrost soil	Hess Creek, Alaska	2067725009$^{\text {e}}$	Meta
Tveit et al. (2013)	Permafrost soil	Ny-Ålesund, Svalbard	SRP014474$^{\text {d}}$	Meta
Tveit et al. (2014)	Permafrost soil	Ny-Ålesund, Svalbard	SRR1509497–SRR1509498, SRR1509518	Meta
			and SRR1509520–SRR1509522$^{\text {d}}$	

$^{\text {a}}$Sanger=Sanger amplicon sequencing, pyrotag=454 pyrotag amplicon sequencing, meta=metagenomics/metatranscriptomics sequencing

$^{\text {b}}$NCBI Genbank database,

$^{\text {c}}$The European Bioinformatics Institute

$^{\text {d}}$NCBI SRA database

$$^{\text {e}}$$IMG/M system

In this contribution, we aim to expand current knowledge on high latitude microbial eukaryote ecosystems by documenting the composition and estimating the abundance of microbial eukaryote communities in four stream sediment samples from Svalbard; two from a glaciated catchment and two from an unglaciated permafrost-dominated catchment, and compare their composition to existing literature data from terrestrial polar environments. We will focus our comparison on the Hamilton et al. (2013) study conducted at Robertson Glacier, Alberta, Canada because they used the same primers, sequencing conditions, and analysis pipeline, enabling a direct comparison.

## Methods

### Fieldsite and sample collection

**Fig. 1:**
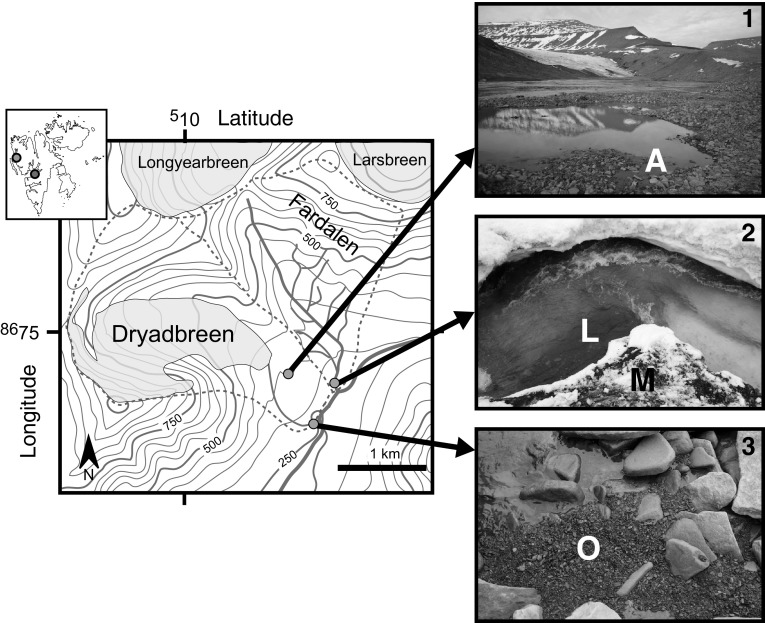
Topographic map of the sediment sampling locations. Contours are displayed at 50 m intervals. The *red dashed lines* demarcate the catchment boundaries. Dryadbreen is on the *left* and Fardalen on the *right*. *Orange circles* mark the locations where sediment samples for eukaryal 18S rRNA gene sequencing were collected. In the unglaciated catchment Fardalen, two samples were collected at the same location. The two locations are illustrated in photograph 2 by the letter of the sample name (*L* and *M*). The two samples collected from the glaciated catchment, Dryadbreen, were collected in different locations. These locations are illustrated by photographs 1 and 3 and the exact location of sampling is shown by the letter of the sample name (*A* or *O*). The *red dot* in the *inset* shows the location of the study area (Latitude, 78$^{\circ }08'$N; Longitude, 15$^{\circ }30'$E) in relation to the rest of Svalbard. The *grey dot* in the *inset* shows the location of Austre Brøggerbreen (Luo et al. 2009). (Color figure online)

Four samples were collected from three different locations in a 1 km$^2$ area, approximately 8 km south-west of Longyearbyen, Svalbard (Fig. 1). Svalbard is located in the Arctic Ocean and has an Arctic climate. In 2012 (the year samples were collected) the mean temperature was −2.0 °C and precipitation was 268 mm, as recorded at Longyearbyen Airport (Nordli et al. 2012).

Two sediment samples were collected from a permafrost-affected valley called Fardalen (Fig. 1), during the beginning of the melt season (18 June 2012). One sediment sample (sample L) was collected from the main stream draining the valley. The sediment was resting on the bottom of the river channel, which was frozen. Water was flowing over the sediment and the pH of this water was 6.2. The second sediment sample (sample M) was collected immediately adjacent to the stream (Fig. 1). This sample was sediment resting on snow.

Two additional samples were collected downstream from the glacier called Dryadbreen in the summer when the majority of the snow-pack had melted. These samples included a sediment sample (sample A) which was collected on 1 August 2012 from a pool of water in the sandur (glacial outwash plain) that was not connected to the main stream, and a river sediment sample (sample O) which was collected on 29 July 2012 immediately adjacent to the main stream, approximately 1 km from the glacier front (Fig. 1).

Sediment samples were scooped directly from the surface into either sterile 300-mL PVC containers or sterile 50-mL centrifuge tubes. No vegetation was observed at any of the sediment collection sites. The samples were stored at ambient temperature (<4 $^{\circ }$C) until they were desiccated by drying at 40 $^{\circ }$C (4 days) to ensure successful transport through customs during international shipment to the USA for molecular analyses. Bacterial 16S rRNA gene diversity data (no archaeal 16S rRNA gene amplicons were recovered) from these sediment samples were previously reported in the context of sulphur and carbon cycling in these catchments (Hindshaw et al. 2016a, b).

### Nucleic acid extraction and quantification

DNA extraction and purification were carried out with a FastDNA Spin Kit for Soil (MP Biomedicals, Solon, OH). DNA was extracted in triplicate from 250 mg subsamples of homogenised sediment. Equal volumes of each replicate extract were pooled and the concentration of DNA was determined using a Qubit dsDNA HS Assay kit (Molecular Probes, Eugene, OR) and a Qubit 2.0 Fluorometer (Invitrogen, Carlsbad, CA). The quantity of DNA extracted per gram of dry weight sediment (gdws) was determined for use in normalising results obtained from downstream qPCR assays (described below).

### PCR amplification and sequencing of eukaryal 18S rRNA genes

For amplification of eukaryote 18S rRNA genes from sediment DNA extracts, primers euk-A7F (5′-AACCTGGTTGATCCTGCCAGT-3′) [modified from Medlin et al. (1988)] and Euk-570R (5′-GCTATTGGAGCTGGAATTAC-3′) (Weekers et al. 1994) were used at an annealing temperature of 42 $^{\circ }$C. These primers target the V1–V3 region of the 18S rRNA gene, corresponding to positions 1–596 of the 18S rRNA gene from *Saccharomyces cerevisiae* (genome accession: RDN18-1 SGD ID: S000006482), and were chosen for use to facilitate direct comparison with results from Hamilton et al. (2013). PCR conditions for amplification of 18S rRNA genes were as previously described by Hamilton et al. (2013).

Eukaryal 18S rRNA gene fragments were barcoded and sequenced using a 454 Genome Sequencer FLX System. Post sequencing processing was performed using the Mothur (ver. 1.31.1) sequence analysis platform (Schloss et al. 2009). Primers were trimmed from sequences, were subjected to a filtering step using the quality scores file to remove sequences with anomalous base calls (Phred threshold of >25 or with greater than eight homopolymers), and remaining sequences were trimmed to a minimum length of 250 bases. Unique sequences were aligned using SILVA databases (release 119) specifying the needleman alignment algorithm and aligned sequences were trimmed using a defined start and end site based on inclusion of 85% of the total sequences; sequences that started before or after these defined positions were removed without further consideration. The resulting unique sequences were pre-clustered with Mothur specifying the single linkage method and two mismatches to remove amplification and sequencing errors. Chimeric sequences were identified and removed using UCHIME (Edgar et al. 2011), as implemented in Mothur. The remaining sequences were clustered and operational taxonomic units (OTUs) were assigned at a sequence similarity of 97% (Stackebrandt and Goebel 1994; Schloss and Handelsman 2006) using the nearest-neighbour method, as implemented in Mothur. The remaining sequences were randomly subsampled to normalise the total number of sequences in each library. A total of 815 18S rRNA gene fragments were subsampled from each of the communities. Representative sequences for each OTU were compiled (representative sequence with the smallest maximum distance to the other sequences) and were classified manually using BLASTn using the NCBI GenBank database. The taxonomic affiliations of OTUs that represented >1.0% of the total sequence reads in any of the seven libraries from Svalbard and Robertson Glacier are reported in Online Resource 1. In addition, a representative sequence from each of the dominant OTUs is provided in Online Resource 2. Raw sequencing data, quality files, and mapping files for eukaryal 18S rRNA genes (Svalbard) are deposited in the NCBI SRA database under accession number SRR1562043.

### Quantification of 18S rRNA genes

The abundance of eukaryal 18S rRNA gene copies per gram dry weight sediment (gdws) was determined via quantitative PCR using a BioRad CFX Connect PCR detection system (Hercules, CA) as previously described (Hamilton et al. 2013). Briefly, qPCR reactions were performed in triplicate with 500 nM forward and reverse primer (described above) and the SsoAdvanced^TM^ Universal SYBR^®^Green Supermix (BioRad) according to the manufacturer’s instructions. The following cycling conditions were used: an initial denaturing at 98 $^{\circ }$C for 30 s followed by 35 cycles of 98 $^{\circ }$C (30 s) and annealing and elongation at 42 $^{\circ }$C (60 s). Specificity of the qPCR assays was verified by melt curve analysis. Control reactions contained no template DNA. Plasmid standards for use in relating template copy number to threshold amplification signals were prepared as previously described (Boyd et al. 2011). qPCR was performed as a first step toward estimating the number of 18S rRNA gene templates as a proxy for the microbial eukaryotic population size in sediment samples collected from Svalbard.

### Phylogenetic diversity calculations

Sequences representing each unique OTU (as defined above) were compiled for each sample. ClustalX (ver. 2.0.9) (Larkin et al. 2007) was then used to align these sequences using default parameters. The eukaryal 18S rRNA gene alignment block was then subjected to evolutionary model prediction via jModeltest (ver. 2.1.1) (Darriba et al. 2012), Maximum-Likelihood phylogenetic reconstruction via PhyML (version 3.0) (Guindon and Gascuel 2003) specifying the general time reversible model and gamma distributed rate variation with a proportion of invariable sites, and rate smoothing using the multidimensional version of Rambaut’s parameterisation as implemented in PAUP (ver. 4.0) (Swofford 2001) as previously described (Meuser et al. 2013).

The rate-smoothed ultrametric tree was used to calculate the Rao quadratic entropy or phylogenetic diversity (Rao PD) for each assemblage with the program Phylocom (ver. 4.0.1) (Webb et al. 2008). Rao’s PD is an abundance weighted metric that describes the pairwise phylogenetic distance between sequences in a community, when compared to the total sequence pool (Rao 1982). Assemblages with higher Rao PD indices exhibit a greater phylogenetic diversity relative to the total sequence pool. Phylocom was also used to calculate Rao’s community phylogenetic dissimilarity for eukaryal assemblages using relative sequence abundance weights and rate-smoothed ultrameric trees. A cluster dendrogram was constructed from the Rao community phylogenetic dissimilarity matrix using PAST (ver. 3.06) (Hammer et al. 2001).

## Results

### Abundance of microbial eukaryote 18S rRNA gene templates

The number of eukaryal 18S rRNA gene copies ranged from 1.62 $\times$ 10$^2$ (Site A) to 6.93 $\times$ 10$^5$ (Site M) templates gdws$^{-1}$ sediment, with an average of 3.2 $\times$ 10$^5$ templates gdws$^{-1}$ sediment (Table 2). This is roughly two orders of magnitude lower than the number of templates detected in subglacial sediments sampled from Robertson Glacier, Canada (Table 2, Hamilton et al. 2013). Although abundances of 18S rRNA gene copies can vary widely in the genomes of microbial eukaryotes (e.g. Gong et al. 2013), it is unlikely that this alone accounts for the two orders of magnitude variation in gene copies. Rather, these results point to the existence of a more robust microbial eukaryote community at Robertson Glacier compared to Svalbard.

### Microbial eukaryote community diversity

Samples A and O from the glaciated catchment had similar richness (138 and 139 OTUs, respectively, Table 2), which was higher than the samples from the unglaciated catchment [106 (L) and 67 (M) OTUs]. The cryoconite sample from Robertson Glacier had greater richness than all four of the Svalbard samples (182 OTUs), snow had similar richness to sample L (103 OTUs) and the subglacial sample had the lowest richness (54 OTUs, Table 2).

The Rao PD was calculated for each of the four sediment eukaryote communities and was similar among samples, ranging from 0.17 to 0.21, with the exception of sample M (0.12) which had a lower diversity (Table 2). The three samples from Robertson Glacier also had similar Rao PD [cryoconite (0.24), snow (0.18), subglacial sediment (0.23)] to the Svalbard communities (with the exception of sample M, Table 2).

**Table 2:** Number of OTUs (proxy for species), estimated sequence coverage, phylogenetic diversity and template abundance associated with 18S rRNA genes sampled from four Svalbard sediment samples and three samples from Robertson Glacier, Canada

Site	Sample	Richness$^{\text {a}}$	Coverage$^{\text {b}}$	Rao PD$^{\text {c}}$	Average templates (1SD) (gdws)	
Svalbard	A	138	0.89	0.21	1.62 $\times$ 10$^{2}$	9.93 $\times$ 10$^{1}$
Svalbard	L	106	0.93	0.20	1.65 $\times$ 10$^{4}$	3.39 $\times$ 10$^{3}$
Svalbard	M	67	0.95	0.12	6.93 $\times$ 10$^{5}$	1.21 $\times$ 10$^{5}$
Svalbard	O	139	0.89	0.17	5.49 $\times$ 10$^{5}$	5.62 $\times$ 10$^{4}$
RG	Sub	54	0.97	0.23	2.18 $\times$ 10$^{7}$	9.83 $\times$ 10$^{5}$
RG	Cryo	182	0.84	0.24	1.68 $\times$ 10$^{7}$	1.68 $\times$ 10$^{6}$
RG	Snow	103	0.92	0.18	6.01 $\times$ 10$^{7}$	6.01 $\times$ 10$^{6}$

Both studies employed the same primers, sequencing conditions and analysis methods

$^{\text {a}}$Richness is measured by the number of unique operational taxonomic units (OTUs) at the 97% sequence similarity

$^{\text {b}}$The percent of the predicted OTU richness, based on rarefaction analysis, that was sampled in the current study

$^\mathbf{c }$Rao’s phylogenetic diversity

**Fig. 2:**
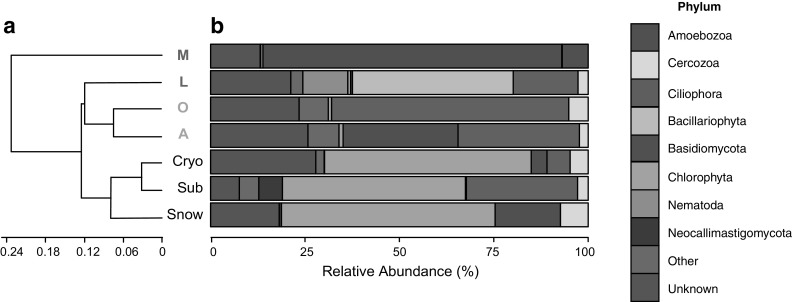
**a** Dendrogram based on the Rao among-community phylogenetic dissimilarity between the eukaryal communities associated with the different sediment samples. Samples *O* and *A* were collected in Dryadbreen (glaciated, *red letters*) and *L* and *M* were collected in Fardalen (unglaciated, *blue letters*). Samples ‘Sub’ (subglacial sediment), ‘Cryo’ (cryoconite) and snow were collected from Robertson Glacier, Canada. Sample *M* is distinct from the rest of the samples which form two clusters matching with the geographical region where the samples were collected. The Cophenetic coefficient for the dendrogram was 0.88. **b** Relative abundance of eukaryal phyla/subkingdoms based on percent identities of 18S rRNA genes to their closest cultivated sequence, as determined by BLASTn analysis. ‘Other’ includes phyla/subkingdoms comprising less than 5% of the total number of sequences (Ascomycota, Blastocladiomycota, Chytridiomycota, Porifera, Rhodophyta, Stramenopiles). (Color figure online)

### Microbial eukaryote community composition

The taxonomic affiliations of 18S rRNA genes recovered from the four Svalbard sediment samples together with the three samples from Robertson Glacier, Canada are depicted in Fig. 2.

Cluster analysis of Rao among-community phylogenetic dissimilarity indicates that the two communities sampled from the glaciated catchment in Svalbard (samples A and O) form a cluster, indicating that they are similar in their composition (Fig. 2). The most abundant phylum associated with these two samples is Ciliophora (32 and 63% of total sequences for A and O, respectively), which are heterotrophic and often bacteriovorus (Lynn 2008). Although the subglacial sediment sample from Robertson glacier also contained a significant amount of Ciliophora (29% of total sequences), the dominant phylum was Chlorophyta (48% of total sequences), which includes phototrophic eukaryotes.

In Sample L from the unglaciated catchment, the dominant sequences (Fig. 2) were closely affiliated with members of the phylum Bacillariophycaea (43% of total sequences), which are autotrophic, followed by sequences affiliated with Ciliophora (17% of total sequences). Sample M was dominated by Basidiomycota (79% of total sequences) and the second most abundant phylum was Amoebozoa (7% of total sequences).

## Discussion

Sample M was the only sample to contain Amoebozoa (7%, Fig. 2), although due to biases in sequencing it may be present in the other samples (Geisen et al. 2015). Amoebozoa have been detected in a snow sample from Vestfold Hills, Antarctica ($\sim$30%, Cameron et al. 2012) and in Svalbard peatlands (12–26% of Protists, Tveit et al. 2013). Unlike the other sediment samples from Svalbard, but similar to the snow sample from Robertson Glacier, sample M also contains no Ciliophora. The most abundant phylum in sample M was Basidiomycota (79%) and snow samples from Thule, Greenland and near the North Pole also contained high relative abundances (>50%) of Fungi (Bachy et al. 2011; Cameron et al. 2015). It should be noted that the microbial eukaryal community composition of snow samples is highly variable (Hamilton et al. 2013; Cameron et al. 2015) and is dependent on local terrestrial (soil) sources (Cameron et al. 2015) and marine microbial aerosols (Harding et al. 2011). Sample M therefore appears to be influenced by the snow it was resting on (Fig. 1) and it is possible that it could be influenced by exogenous eukarya transported or deposited by the snow.

Similarities were observed between Sample L and a cobble-bottomed headwater stream on the North Slope of Alaska which also drains an unglaciated area underlain by continuous permafrost (Crump et al. 2012). Similar to Sample L (Fig. 2), the latter site contained high abundances of Bacillariophyceae (Stramenopiles) (25%) and Ciliophora (13%), however, Chytridiomycota (Fungi, 32%) was also detected at this site whereas no fungal sequences were detected in Sample L.

Ciliophora was the most abundant phylum in the two samples from the glaciated catchment [(63% (Sample O) and 32% (Sample A)] and the second most abundant phylum in Sample L (17%). High abundances of Ciliophora were observed in porewater near Toolik Lake (40%, Crump et al. 2012) and high relative abundances (>45%) of the superphylum Alveolata have additionally been reported in permafrost soil samples (Mackelprang et al. 2011; Tveit et al. 2013; Jansson and Taş 2014) and glacial meltwater (Luo et al. 2009). The relative proportion of Ciliophora in a sample inferred from 18S-rRNA gene sequencing can be greater than their actual abundance since genomes can encode for multiple copies of this gene (Gong et al. 2013). However, a study in Arctic peat soils using metatranscriptomics (Geisen et al. 2015) also found a high relative abundance ($\sim$48%) of Ciliophora. This suggests that Ciliophora, which are predominantly heterotrophic and often bacterivorous, may indeed be a common feature of high-latitude soils and sediments with high moisture and high organic matter content (Tveit et al. 2013; Geisen et al. 2015).

**Fig. 3:**
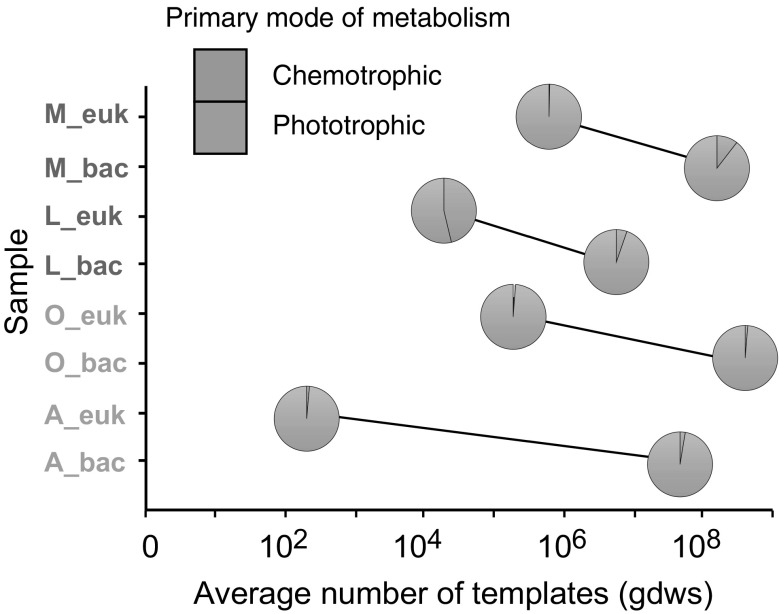
The average number of templates, which is a proxy of the population size, for the microbial eukaryote and bacterial communities in each of the samples. The two communities for each sample are connected by a *line*. Each point is illustrated by a pie chart which depicts the relative abundance of bacteria (bac) or microbial eukaryotes (euk) inferred to be phototrophic or chemotrophic based on percent identities of 16S rRNA or 18S rRNA genes, respectively, to their closest cultivated sequence, as determined by BLASTn analysis. Samples *O* and *A* were collected in Dryadbreen (glaciated, *red letters*) and *L* and *M* were collected in Fardalen (unglaciated, *blue letters*). Bacteria data are taken from (Hindshaw et al. 2016a) and the calculation of the relative abundance of phototrophic and chemotrophic bacteria is described in Online Resource 3. (Color figure online)

The two sediment samples from the glaciated catchment together with Sample M from the unglaciated catchment have low relative abundances of sequences affiliated with phyla known to contain phototrophic eukaryotes (<2%, Bacillariophyta, Chlorophyta and Stramenopiles, Fig. 3). Chlorophytes, which are phototrophic algae, also formed a minor component of a glacial meltwater sample from Svalbard (8%, Luo et al. 2009) and an Alaskan headwater stream sample (5%, Crump et al. 2012). The presence of Bacillariophyta (diatoms) in sample L may reflect the lower suspended sediment concentrations of the unglaciated stream, compared to the stream draining the glaciated catchment (Fig. 1), allowing greater light penetration.

The low relative abundance of phototrophic microbial eukaryotes in the glaciated catchment supports the notion that microbial eukaryotes in this environment are predominantly heterotrophic (chemotrophic) and are dependent on bacterial/archaeal biomass, or relict organic carbon for energy generation. Moreover, a calculation of the prevalence of phototrophy amongst the bacterial communities in these sediment samples (Online Resource 3 and Hindshaw et al. 2016a) suggests that more than 90% of the bacterial species identified are chemotrophic (Fig. 3). Taken together this suggests that the microbial systems in Svalbard, particularly those from the glaciated catchment, appear to be dominated by chemotrophy. More data would be required to assess how widespread chemotrophy is in high-latitude environments.

Surprisingly, given the lack of light, the Robertson Glacier subglacial sediment sample contains a high relative abundance (48%) of chlorophyta, but it is possible that this sample site was influenced by microbial eukaryotes washed down moulins and crevasses (which connect the surface to the subsurface of a glacier) from cryoconite and snow which both contain similar relative abundances (55–57%, respectively) of chlorophyta. Robertson Glacier is an alpine glacier with an established hydrological connection between the surface and the bed of the glacier during the melt season (Sharp et al. 2002). In contrast, the thermal regimes of Dryadbreen (this study) and Austre Brøggerbreen (Luo et al. 2009) are expected to be cold-based with temperate patches, based on similar sized glaciers in the same area (Etzelmüller et al. 2000; Etzelmüller and Hagen 2005). Under this thermal regime, limited supra-glacial water is expected to reach the bed of the glacier (Irvine-Fynn et al. 2011), restricting the influx of microbial eukaryotes from the surface. Therefore, the relative abundance of phototrophic microbial eukaryotes present in, and downstream of, subglacial environments may be a function of the type of glacier (cold-based versus temperate) which affects how much surface water reaches the bed of the glacier.

Alternatively, it has recently been shown that geology has a strong influence on bacterial communities (Mitchell et al. 2013) and presumably therefore on microbial eukaryote communities. Dryadbreen is underlain by shale, siltstone and sandstone (Hindshaw et al. 2016a), whereas Robertson Glacier is predominantly underlain by carbonate rocks, although minor shale, siltstone and sandstone are present (Sharp et al. 2002). This difference in geology may lead to the observed difference in the microbial eukaryote community composition between the two sites. Perhaps the greater abundance of shale, which contains pyrite (FeS$_{2}$) and organic matter, in the Svalbard catchments may favour chemotrophic bacteria utilising iron and sulphur redox reactions for energy and heterotrophic organisms utilising the relict organic matter.

## Conclusions

Four sediment samples collected in a 1 km$^{2}$ area from Svalbard had dissimilar microbial eukaryote community compositions. All sites had similar lithology and meteorological conditions, but two of the samples were from an unglaciated permafrost-dominated catchment and two were from a glaciated catchment. A river sediment sample from the unglaciated catchment had similar composition at the phylum level to a sample previously described from an Alaskan headwater stream, suggesting similarities among streams draining permafrost. However, there were major differences between the samples from the glaciated catchment and those from Robertson Glacier, Canada, despite both sites being glaciated. The major difference was the relative abundance of microbial eukaryotes inferred to be phototrophic or heterotrophic (chemotrophic) and may arise due to differences in geology, glacial hydrology or a combination of environmental factors. Further research would be required to elucidate which environmental factors are most important in shaping the microbial eukaryote community, providing insight as to how these high-latitude communities and their impact on nutrient cycling may be affected by a changing climate.

## Electronic supplementary material

Below is the link to the electronic supplementary material.

Supplementary material 1 (pdf 0 KB)

Supplementary material 2 (pdf 0 KB)

Supplementary material 3 (pdf 0 KB)
