# Supplementary material for: Diversity and abundance of microbial eukaryotes in stream sediments from Svalbard
Source: Polar Biol. 2017 Mar 31;40(9):1835–43. doi: 10.1007/s00300-017-2106-3 (PMC6961512; doi:10.1007/s00300-017-2106-3)
Supplement: Supplementary file 1 — Supplementary material 1 (pdf 0 KB) [file 300_2017_2106_MOESM1_ESM.pdf]

Online Resource 1. Taxonomic affiliations and relative abundances of 18S rRNA gene OTUs in samples A, L, M, and O (Svalbard) and Sub, Snow and Cryo (Robertson Glacier, Canada) based on BLASTn analysis using the NCBI GenBank database. Only OTUs that represented >1.0% of the relative abundance of 18S rRNA genes in any of the 7 samples were subjected to BLAST analysis. Raw sequencing data, quality files, and mapping files for eukaryal 18S rRNA genes (Svalbard) are deposited in the NCBI SRA database under accession number SRR1562043.

OTU	Sample Designation							Taxonomic Rank (Based on closest BLASTn match)			% Identity
	Svalbard				Robertson						
	A	L	M	O	Sub	Snow	Cryo	Phylum	Order	Genus	
Otu0024	0.0	0.0	0.1	5.9	0.0	0.0	0.0	Alveolata	Sporadotrichida	Oxytricha	99
Otu0036	0.0	0.0	0.0	2.9	0.0	0.0	0.0	Alveolata	Sporadotrichida	Meseres	98
Otu0028	2.0	0.0	0.0	1.2	0.0	0.0	0.0	Alveolata	Sporadotrichida	Orthamphisiella	99
Otu0007	0.0	0.0	0.0	39.3	0.0	0.0	0.2	Alveolata	Stichotrichida	Pseudouroleptus	98
Otu0004	7.4	17.2	0.0	11.5	29.4	0.0	3.7	Alveolata	Stichotrichida	Orthamphisiella	100
Otu0006	22.8	0.0	0.0	0.1	0.0	0.0	0.0	Alveolata	Stichotrichida	Oxytricha	99
Otu0042	0.0	0.0	0.0	0.0	0.0	0.0	2.2	Alveolata	Urostylida	Anteholosticha	98
Otu0037	0.0	0.0	0.0	1.8	0.0	0.0	0.0	Alveolata	***No rank***	Parabistichella	98
Otu0005	0.0	0.0	6.9	0.0	0.0	0.0	0.0	Amoebozoa	***No rank***	Flamella	90
Otu0056	0.0	0.0	0.0	0.0	3.3	0.0	0.0	Ascomycota	Hypocreales	Verticillium	100
Otu0034	1.5	0.0	0.0	0.0	0.0	0.6	0.1	Ascomycota	Lecanorales	Bellemerea	100
Otu0026	1.2	0.0	0.0	3.6	0.0	0.0	0.0	Ascomycota	Pezizales	Ascodesmis	91
Otu0008	0.0	42.6	0.2	0.0	0.0	0.0	0.0	Bacillariophyta	Fragilariales	Diatoma	100
Otu0014	0.4	0.0	2.9	0.0	0.0	0.0	0.0	Basidiomycota	Cystofilobasidiales	Mrakia	100
Otu0003	30.1	0.5	0.0	0.0	0.0	0.0	0.0	Basidiomycota	Entylomatales	Tilletiopsis	96
Otu0022	0.0	0.0	0.0	0.0	0.0	4.7	0.0	Basidiomycota	Kriegeriales	Kriegeria	99
Otu0009	0.0	0.0	2.8	0.0	0.2	0.1	0.0	Basidiomycota	Kriegeriales	Kriegeria	99
Otu0001	0.0	0.0	67.2	0.0	0.0	0.0	0.1	Basidiomycota	Leucosporidiales	Leucosporidium	100
Otu0015	0.0	0.0	0.0	0.0	0.0	9.4	4.0	Basidiomycota	Leucosporidiales	Leucosporidium	96
Otu0010	0.0	0.0	3.8	0.0	0.0	0.0	0.0	Basidiomycota	Leucosporidiales	Leucosporidium	96

Otu0016	0.0	0.0	2.1	0.0	0.0	0.1	0.0	Basidiomycota	Leucosporidiales	Leucosporidium	99
Otu0040	0.0	0.0	0.0	0.0	0.0	1.8	0.0	Basidiomycota	Leucosporidiales	Leucosporidium	98
Otu0043	0.0	0.0	0.0	0.0	0.0	1.1	0.0	Basidiomycota	Leucosporidiales	Leucosporidium	98
Otu0020	5.3	0.0	0.0	2.5	0.0	0.0	0.0	Blastocladiomycota	Blastocladiales	Paraphysoderma	85
Otu0029	0.5	0.4	0.0	0.6	0.0	0.6	1.2	Cercozoa	Cercomonadida	Bodomorpha	99
Otu0017	1.8	0.1	0.0	2.7	0.0	6.0	2.3	Cercozoa	Cercomonadida	Bodomorpha	98
Otu0068	0.0	0.0	0.0	0.0	2.3	0.0	0.1	Cercozoa	Cercomonadida	Bodomorpha	97
Otu0038	0.0	0.0	0.0	1.8	0.0	0.7	0.1	Cercozoa	Cercomonadida	Bodomorpha	98
Otu0048	0.0	2.2	0.0	0.0	0.0	0.0	0.0	Cercozoa	Vampyrellida	Arachnula	92
Otu0081	0.0	0.0	0.0	0.0	0.5	0.0	1.0	Cercozoa	***No rank***	Rhogostoma	95
Otu0002	0.1	0.0	0.0	0.0	0.0	45.5	4.3	Chlorophyta	Chlamydomonadales	Chlamydomonas	99
Otu0012	0.0	0.0	0.0	0.1	6.4	0.7	18.7	Chlorophyta	Chlamydomonadales	Chloromonas	99
Otu0011	0.0	0.0	0.0	0.0	16.0	0.0	14.4	Chlorophyta	Chlamydomonadales	Chlorococcum	99
Otu0019	0.0	0.0	0.0	0.0	0.2	9.1	0.1	Chlorophyta	Chlamydomonadales	Chloromonas	98
Otu0059	0.0	0.0	0.0	0.0	0.0	1.2	0.2	Chlorophyta	Chlamydomonadales	Chlamydomonas	99
Otu0090	0.0	0.0	0.0	0.0	0.2	0.0	1.0	Chlorophyta	Chlamydomonadales	Chloromonas	99
Otu0065	0.0	0.0	0.0	0.0	0.0	0.0	1.3	Chlorophyta	Chlorellales	Micractinium	100
Otu0018	1.0	0.4	0.0	0.6	13.7	0.0	8.2	Chlorophyta	Microthamniales	Stichococcus	100
Otu0021	0.0	0.0	0.0	0.1	9.9	0.0	5.8	Chlorophyta	Ulotrichales	Pseudendocloniopsis	100
Otu0041	0.0	0.4	0.0	0.0	2.0	0.0	0.7	Chlorophyta	Ulotrichales	Pseudendocloniopsis	99
Otu0083	0.0	0.0	0.0	0.0	0.0	0.0	1.1	Chytridiomycota	Chytridiales	Chytridium	93
Otu0072	0.0	0.0	0.0	1.3	0.0	0.0	0.0	Chytridiomycota	Monoblepharidales	Monoblepharella	94
Otu0013	0.0	11.9	0.6	0.0	0.0	0.0	0.0	Nematoda	Rhabditida	Cylicostephanus	91
Otu0033	0.0	0.0	0.0	0.0	6.3	0.0	0.1	Neocallimastigomycota	Neocallimastigales	Neocallimastix	96
Otu0069	0.0	0.2	0.0	0.0	0.0	0.0	1.0	Porifera	Suberitida	Halichondria	89
Otu0084	0.0	0.0	0.0	0.0	1.8	0.0	0.0	Rhodophyta	Batrachospermales	Kumanoa	97
Otu0055	0.2	1.5	0.1	0.1	0.0	0.0	0.0	Stramenopiles	Hydrurales	Hydrurus	99
Otu0054	0.0	1.5	0.0	0.2	0.0	0.0	0.0	Stramenopiles	Hydrurales	Hydrurus	99
