# Supplementary material for: Diversity and abundance of microbial eukaryotes in stream sediments from Svalbard
Source: Polar Biol. 2017 Mar 31;40(9):1835–43. doi: 10.1007/s00300-017-2106-3 (PMC6961512; doi:10.1007/s00300-017-2106-3)
Supplement: Supplementary file 2 — Supplementary material 2 (pdf 0 KB) [file 300_2017_2106_MOESM3_ESM.pdf]

**Online Resource 3: Quantification of Phototrophic Bacteria**

Phototrophic bacteria are known to belong to the following groups: Cyanobacteria, Green sulfur bacteria (Chorobiacaea), Purple non sulfur bacteria (Chromatiacaea and Ectothiorhodospiraceae), Acidiobacteria, Heliobacteria and Filamentous anoxygenic photoautotrophs (FAP, Chloroflexi). It is acknowledged that not all members of these groups are phototrophic but as a conservative estimate, which may overestimate the abundance of phototrophs, we assume all members are phototrophic.

Sequences associated with Cyanobacteria, Green sulfur bacteria and Heliobacteria were not recovered from any of the samples. We therefore sum all sequences associated with Purple non sulfur bacteria (Chromatiales), Acidobacteria and FAP (Chloroflexi). The relative abundance of OTUs associated with phototrophic bacteria is given in Table 1. The calculation for the percentage of microbial eukaryotes inferred to be phototrophic is described in the main manuscript.

**Table 1**: Percentage of bacteria and microbial eukaryotes inferred to be phototrophic.

| Sample | % Phototrophs | |
|---|---|---|
| | Bacteria | Microbial eukaryotes |
| A | 2.6 | 1.3 |
| L | 5.3 | 46.3 |
| M | 10.5 | 0.4 |
| O | 1.3 | 1.2 |

The abundance of bacteria (Table 2) relative to microbial eukaryotes can be assessed by comparing the average number of templates recovered. The average number of templates recovered for bacteria is around 2 orders of magnitude greater than the average number of templates for microbial eukaryotes at a given site. All four sites are therefore inferred to be dominantly chemotrophic

**Table 2**: Template abundance of 16S rRNA genes.

| Sample | Average Templates (1SD) (gdws) | |
|---|---|---|
| A | $4.25 \times 10^7$ | $6.49 \times 10^6$ |
| L | $5.21 \times 10^6$ | $3.69 \times 10^5$ |
| M | $1.41 \times 10^8$ | $1.58 \times 10^7$ |
| O | $4.63 \times 10^8$ | $5.01 \times 10^7$ |

.
