# Supplementary material for: Diversity and abundance of microbial eukaryotes in stream sediments from Svalbard
Source: Polar Biol. 2017 Mar 31;40(9):1835–43. doi: 10.1007/s00300-017-2106-3 (PMC6961512; doi:10.1007/s00300-017-2106-3)
Supplement: Supplementary file 3 — Supplementary material 3 (pdf 0 KB) [file 300_2017_2106_MOESM2_ESM.pdf]

Online Resource 2. Representative sequence for each of the operational taxonomic units described in Online Resource 1.

OTU	**Representative Sequence**
Otu0024	GTCATATGCTTGTCTCAAAGACTAAGCCATGCATGTCTAAGTATAAATGTTATACAGTGAAACTGCGAATGGCTCATTAAAACAGTTATAGTTTATTTGATAATCAAACTTACATGGATAACCGTGGTA<br>ATTCTAGAGCTAATACATGCTGGTTAGCCTGACTTTTAGGAAGGGCTGTATTTATTAGATAACAAACCAATATTCCCCGTGTCTATTGTGACGACTCATAATAACTGATCGAATCG
Otu0036	GTCATATGCTTGTCTCAAAGACTAAGCCATGCATGTCTAAGTATGAATGTTATACAGTGAAACTGCGAATGGCTCATTAAAACAGTTATTGTTTATTTGATAATCGAATTTTACATGGATAACCGTGGTA<br>ATTCTAGAGCTAATACATGCTGTTAAGCCTGACTTTTTGGAAGGGCTGTATTTATTAGATAACAAACCAATATTCCTTGTGTCTATTGTGATGACTCATAATAACTGATCGAATCG
Otu0028	GTCATATGCTTGTCTCAAAGACTAAGCCATGCATGTCTAAGTATAAATGTTATACAGTGAAACTGCGAATGGCTCATTAAAACAGTTATAGTTTATTTGATAATCGAATCTACATGGATAACCGTGGTA<br>ATTCTAGAGCTAATACATGCGGCTACGCCTGACTCTCGAGGAAGGGCGGTATTTATTAGATAACAAACCAATATTCCCTGTGTCTATTGTGATGACTCATAGTAACTGATCGAATCG
Otu0007	GTCATATGCTTGTCTCAAAGACTAAGCCATGCATGTCTAAGTATGAATGTTATACAGTGAAACTGCGAATGGCTCATTAAAACAGTTATTGTTTATTTGATAATCAAATTTACATGGATAACCGTGGTA<br>ATTCTAGAGCTAATACATGCTGATATGCCTGACTCTTAGGAAGGGCTGTATTTATTAGATAACAAACCAATATTCCTCGTGTCTATTGTGATGACTCATAATAACTGATCGAATCG
Otu0004	GTCATATGCTTGTCTCAAAGACTAAGCCATGCATGTCTAAGTATAAATGTTATACAGTGAAACTGCGAATGGCTCATTAAAACAGTTATAGTTTATTTGATAATCGAATTTACATGGATAACCGTGGTA<br>ATTCTAGAGCTAATACATGCTGGTTAGCCTGACTTTTGAGGAAGGGCTGTATTTATTAGATAACAAACCAATATTCCTTGTGTCTATTGTGATGACTCATAATAACTGATCGAATCG
Otu0006	GTCATATGCTTGTCTCAAAGACTAAGCCATGCATGTCTAAGTATAAATGTTATACAGTGAAACTGCGAATGGCTCATTAAAACAGTTATAGTTTATTTGATAATCAAACTTACATGGATAACCGTGGTA<br>ATTCTAGAGCTAATACATGCTGTTAAGCCTGACTTTTAGGAAGGGCTGTATTTATTAGATAACAAACCAATATTCCTTGTGTCTATTGTGATGACTCATAATAACTGATCGAATCG
Otu0042	GTCATATGCTTGTCTCAAAGACTAAGCCATGCATGTCTAAGTATGAATGATATACAGTGAAACTGCGAATGGCTCATTAAAACAGTTATAGTTTATTTGATAATCAAATTTACATGGATAACCGTGGTA<br>ATTCTAGAGCTAATACATGCTGTTTGGCCTGACTTTTAGGAAGGGCCGTATTTATTAGATAACAAACCAATATTCCTCGTGTCTATTGTGACGACTCATAATAACTGATCGAATCG
Otu0037	GTCATATGCTTGTCTCAAAGACTAAGCCATGCATGTCTAAGTATAAATGTTATACAGTGAAACTGCGAATGGCTCATTAAAACAGTTATAGTTTATTTGATAATCGAATTTTACATGGATAACCGTGGT<br>AATTCTAGAGCTAATACATGCTGGTTCGCCTGACTCTCGAGGAAGGGCGGTATTTATTAGATAACAAACCAATATTCCCCCGTGTCTATTGTGATGACTCATAATAACTGATCGAATCG
Otu0005	GTCATATGCTTGTCTCAAAGATTAAGCCATGCATGTCTTAGTTTAAGTATTAAACAGCGAAACTGCGAACGGCTCATTAAATCAGTTATGACTTACCTGAATTATTCTTTTACTCGGATAACTGTAGTAA<br>TTCTAGAGCTAATACGTGCTTTATGACCTATCGAGCAATCGGTGGGCGCACTTGTTAGATTCTATAACCCATCCCTTCGGGGAAAAAATGATTAATAACAAATGACCGCAGACCG
Otu0056	GTCATATGCTTGTCTCAAAGATTAAGCCATGCATGTCTAAGTATAAGCAATTACAAAGCGAAACTGCGAATGGCTCATTATATAAGTTATCGTTTATTTGATAGTACCTTACTACATGGATAACCGTGGT<br>AATTCTAGAGCTAATACATGCTGAAAATCCCGACTTCGGAAGGGATGTATTTATTAGATACAAAACCAATGCCCTTCGGGGCTCTCTTGGTGATTCATGATAACTTCTCGAATCG
Otu0034	GTCATATGCTTGTCTCAAAGATTAAGCCATGCATGTCTAAGTATAAGCAATCTATACGGTGAAACTGCGAATGGCTCATTAAATCAGTTATCGTTTATTTGATAGTACCTTACTACTTGGATAACCGTGG<br>TAATTCTAGAGCTAATACATGCTAAAAACCTCGACTTCGGAAGGGGTGTATTTATTAGATAAAAAACCAATGCCCTTCGGGGCTCCTTGGTGATTCATAATAACTTAACGAATCG
Otu0026	GTCATATGCTTGTCTCAAAGATTAAGCCATGCATGTCTAAGTATAGCAATATACAGTGAAACTGCGAATGGCTCATTATATCAGTAATAGTTTATTTGATAGTACCTTACTACATGGATAACCGTAGTAA<br>TTCTAGAGCTAATACATGCGCAAAATCCCGACTTTTCGGAAGGGATGTATTTATTAGATCAAAAACCAACCTGGCTTCGGCCTTGACACTTGGTGATTCATAATAACTGATCGAATCG
Otu0008	GTCATACGCTCGTCTCAAAGATTAAGCCATGCATGTCTAAGTATAAATCTTTTACTTTGAAACTGCGAACGGCTCATTATATCAGTTATAGTTTATTTGATAGTCCCTTACTACTTGGATAACCGTAGTAA<br>TTCTAGAGCTAATACATGCGTCAATACCCTTCTGGGGTAGTATTTATTAGATTGAAACCAACCGCTTCGGCGTGATGTGGTGATTCATAATAAATTTGCGAATCG
Otu0014	GTCATATGCTTGTCTCAAAGATTAAGCCATGCATGTCTAAGTATAAACAAATTTTTACTGTGAAACTGCGAATGGCTCATTAAATCAGTTATAGTTTATTTGATGGTACCTTGCTACATGGATAACTGTG<br>GTAATTCTAGAGCTAATACATGCTTAAAAGCCCCAACTTCTGGAAGGGGTGTATTTATTAGATAAAAAACCAACGTGGGAAACCACTCCTTTGGTGATTCATAATAACTTCTCGAATCG
Otu0003	GTCATATGCTTGTCTCAAAGATTAAGCCATGCATGTCTAAGTATAAACACGTTATACTGTGAAACTGCGAATGGCTCATTAAATCAGTTATAGTTTATTTGATGGTTTTTGCTACATGGATAACTGTGGT

	AATTCTAGAGCTAATACATGCGTACAAGCCCCGACTTCTGGAAGGGGTGTATTTATTAGATAAAAAATCAACACTGTTGGTGAATCATGATAACTTCTCGGATCG
Otu0022	AGTCATATGCTTGTCTCAAAGATTAAGCCATGCATGTCTAAGTTTAAGCAATAAACGGTGAAACTGCGAATGGCTCATTAAATCAGTCATAGTTTATTTGATGGTACCCTACTACATGGATAACTGTGG<br>TAATTCTAGAGCTAATACATGCCGAAAAATCTCGACTTCTGGAAGAGATGTATTTATTAGATCCAAAACCAATGGCCTTCGGGTCCCCTACGGTGAATCATGATAACTGCTCGAATCG
Otu0009	GTCATATGCTTGTCTCAAAGATTAAGCCATGCATGTCTAAGTTTAAGCAATAAACGGTGAAACTGCGAATGGCTCATTAAATCAGTCATAGTTTATTTGATGGTACCCTACTACATGGATAACTGTGGT<br>AATTCTAGAGCTAATACATGCCGAAAAATCTCGACTTCTGGAAGAGATGTATTTATTAGATCCAAAACCAATGGCCTCCGGGTCTCCTTGGTGAATCATGATAACTGCTCGAATCG
Otu0001	GTCATATGCTTGTCTCAAAGATTAAGCCATGCATGTCTAAGTTTAAGCAATAAACGGTGAAACTGCGAATGGCTCATTAAATCAGTCATAGTTTATTTGATGGTACCCTACTACATGGATAACTGTGGT<br>AATTCTAGAGCTAATACATGCCGAAAAATCTCGACTTCTGGAAGAGATGTATTTATTAGATCCAAAACCAGTGGCCTTCGGGTCTCCTTGGTGAATCATGATAACTGCTCGAATCG
Otu0015	AGTCATATGCTTGTCTCAAAGATTAAGCCATGCATGTCTAAGTTTAAGCAATAAACGGTGAAACTGCGAATGGCTCATTAAATCAGTCATAGTTTATTTGATGGTACCCTACTACATGGATAACTGTGG<br>TAATTCTAGAGCTAATACATGCCGAAAAATCTCGACTTCTGGAAGAGATGTATTTATTAGATCCAAAGCCAATGGCGGCAACGTCTTTAGGTGAATCATGATAACTGCTCGAATCG
Otu0010	GTCATATGCTTGTCTCAAAGATTAAGCCATGCATGTCTAAGTATAAACAAATTTTTACTGTGAAACTGCGAATGGCTCATTAAATCAGTTATAGTTTATTTGATGGTACCCTACTACATGGATAACTGTG<br>GTAATTCTAGAGCTAATACATGCCGAAAAATCTCGACTTCTGGAAGAGATGTATTTATTAGATCCAAAACCAGTGGCCTTCGGGTCTCCTTGGTGAATCATGATAACTGCTCGAATCG
Otu0016	GTCATATGCTTGTCTCAAAGATTAAGCCATGCATGTCTAAGTTTAAGCAATAAACGGTGAAACTGCGAATGGCTCATTAAATCAGTCATAGTTTATTTGATGGTACCCTACTACATGGATAACTGTGGT<br>AATTCTAGAGCTAATACATGCCGAAAAATCTCGACTTCTGGAAGAGATGTATTTATTAGATCCAAAACCATGGCCTTCGGGTCTCCTTGGTGAATCATGATAACTGCTCGAATCG
Otu0040	GTCATATGCTTGTCTCAAAGATTAAGCCATGCATGTCTAAGTTTAAGCAATAAACGGTGAAACTGCGAATGGCTCATTAAATCAGTCATAGTTTATTTGATGGTACCCTACTACATGGATAACTGTGGT<br>AATTCTAGAGCTAATACATGCCGAAAAATCTCGACTTCTGGAAGAGATGTATTTATTAGATCCAAAGCCAGCGGCCGCAAGGTCTCCTTGGTGAATCATGATAACTGCTCGAATCG
Otu0043	AGTCATATGCTTGTCTCAAAGATTAAGCCATGCATGTCTAAGTTTAAGCAATAAACGGTGAAACTGCGAATGGCTCATTAAATCAGTCATAGTTTATTTGATGGTACCCTACTACATGGATAACTGTGG<br>TAATTCTAGAGCTAATACATGCCGAAAAATCTCGACTTCTGGAAGAGATGTATTTATTAGATCCAAAACCAATGGCCTTCGGTCCCCGTACGGTGAATCATGATAACTAGCTCGAATCG
Otu0020	GTCATATGCTTGTCTAAAGGACTAAGCCATGCATGTCTAAGTATAAGCAATTTATACTTGTGAAACTGCGAATGGCTCATTAAATCAGTTATAGTTTATTTGATATTTTCTTGCTACATGGATACCGGTG<br>GCAAAGTACCGCTAATACATGCAAACAATCCCGCAAGGGATGTATTTATTAGATACGCAAACCAACCCGGGCAACCGGAGTATGCTGACTCATAATAACTTCACGAATCG
Otu0029	GTCATATGCTTGTCTCAAAGATTAAGCCATGCATGTCTAAGTATAAACATCTTTATACAGTGAAACTGCGTACAGCTCATTATATCAGTTATTATTTATTTGATGGTACCTTACTACATGGATACCCGTAG<br>TAATTCTAGAGCTAATACATGCGTCAAGTCCCGACTTTTTGGAAGGGATGTATTTATTAGATAAAAACCAATGCAGGCAACTGCTCTCTTGGTGATTCATAGTAACTTTTCGGATCG
Otu0017	GTCATATGCTTGTCTCAAAGATTAAGCCATGCATGTCTAAGTATAAACATCTTTATACAGTGAAACTGCGTACAGCTCATTATATCAGTTATTATTTATTTGATGGTACCTTACTACATGGATACCCGTAG<br>TAATTCTAGAGCTAATACATGCGTCAAGTCCCGACTCTTTGGAAGGGATGTATTTATTAGATAAAAACCATGCAGGCAACTGCTCTCTTGGTGATTCATAGTAACTTTAACGGATCG
Otu0068	GTCAGATGCTTGTCTCAAAGATTAAGCCATGCATGTCTAAGTATAAACATCTTTATACAGTGAAACTACGTACAGCTCATTATATCAGTTATTATTTATTTGATGGTACCTTACTACATGGATACCCGTA<br>GTAATTCTAGAGCTAATACATGCGTAAAGCCCCGATTTTTGGAAGGATGTATTTATTAGATAAAAACCAATGCAGGCAACTGCTCTCTTGGTGATTCATAGTAACTTTAACGGATCG
Otu0038	GTCATATGCTTGTCTCAAAGATTAAGCCATGCATGTCTAAGTATAAACATCTTTATACAGTGAAACTGCGTACAGCTCATTATATCAGTTATTATTTATTTGATGGTACCTTACTACATGGATACCCGTAG<br>TAATTCTAGAGCTAATACATGCGTCAAGTCCCGACTCTTTGGAAGGGATGTATTTATTAGATAAAAACCATGCAGGTAACTGCTCTCTTGGTGATTCATAGTAACTTTAACGGATCG
Otu0048	GTCATATGCTTGTCTCAAAGATTAAGCCATGCAAGTCTCTGTATAAGCATTTATACTGTGAAACTGCGGAAAGCTCATTATATCAGTTATAGTTTATTTGATGGTACCCCTTACTACATGGATAACCGTA<br>GTAATTCTAGAGCTAATACATGCCCAGAAAGCCCGACTTCGGAAGGGCCGGACTTATTAGATCAAAGAGCCAACCCTCCTCGGAGGACTAAGCTGAATCATAGTAATTTTGCTAATCG
Otu0081	GTCATATGCTTGTCTCAAAGATTAAGCCATGCATGTCTAAGTATAAACAACTTTATACTGTAAAACTGCGAACGGCTCATTATATCAGCAATAATTTATTTGATGATTTCTTACTACATGGATAACTGTA<br>GTAATTCTAGAGCTAATACATGCGTAAAGTCCCAACTCTTGCGGGAAGGGATGTATTTATTAGATAAAAACCAATGCGGGTTCTGCTCGCCTTTGTGTTGAATCATAGTAACC
Otu0002	GTCATATGCTTGTCTCAAAGATTAAGCCATGCATGTCTAAGTATAAACTGCTTTATACTGTGAAACTGCGAATGGCTCATTAAATCAGTTATAGTTTATTTGATGGTACCTACTACTCGGATAACCGTAG<br>TAATTCTAGAGCTAATACGTGCGTAAATCCCGACTTCTGGAAGGGACGTATTTATTAGATAAAAGGCCAGCCGGACTCTGTCCGACCTGCGGTGAATCATGATAACTTCACGAATCG

Otu0012	GTCATATGCTTGTCTCAAAGATTAAGCCATGCATGTCTAAGTATAAACTGCTTTATACTGTGAAACTGCGAATGGCTCATTAAATCAGTTATAGTTTATTTGATGGTACTTACTACTCGGATAACCGTAGTAATTCTAGAGCTAATACGTGCGTAAATCCCGACTTCTGGAAGGGACGTATTTATTAGATAAAAGGCCAGCCGGGCTTGCCCGACTTTAGGTGAATCATGATAACTCCACGAATCG
Otu0011	GTCATATGCTTGTCTCAAAGATTAAGCCATGCATGTCTAAGTATAAACTGCTTATACGGTGAAACTGCGAATGGCTCATTAAATCAGTTATAGTTTATTTGATGGTACTTTTACTCGGATAACCGTAGTAATTCTAGAGCTAATACGTGCGTAAATCCCGACTTATGGAAGGGACGTATTTATTAGATAAAAGGCCAGCCGGGCTTGCCCGAACTTAGGCGAATCATGATAACTTCACGAATCG
Otu0019	GTCATATGCTTGTCTCAAAGATTAAGCCATGCATGTCTAAGTATAAACTGCTTTATACTGTGAAACTGCGAATGGCTCATTAAATCAGTTATAGTTTATTTGATGGTACTTACTACTCGGATAACCGTAGTAATTCTAGAGCTAATACGTGCGTAAATCCCGACTCTTGGAAGGGACGTATTTATTAGATAAAAGGCCAGCCGTGCTTGCACGACTTTAGGTGAATCATGATAACTCCACGAATCG
Otu0059	GTCATATGCTTGTCTCAAAGATTAAGCCATGCATGTCTAAGTATAAACTGCTTTATACTGTGAAACTGCGAATGGCTCATTAAATCAGTTATAGTTTATTTGATGGTACCTACTACTCGGATAACCGTAGTAATTCTAGAGCTAATACGTGCGTAAATCCCGTCTTCTGGAAGGGACGTATTTATTAGATAAAAGGCCAGCCGGACTCTGTCCGACCTGCGGTGAATCATGATAACTTCACGAATCG
Otu0090	GTCATATGCTTGTCTCAAAGATTAAGCCATGCATGTCTAAGTATAAACTGCTTTATACTGTGAAACTGCGAATGGCTCATTAAATCAGTTATAGTTTATTTGATGGTACTTACTACTCGGATAACCGTAGTAATTCTAGAGCTAATACGTGCGTAAATCCCGACTTCTGGAAGGGACGTATTTATTAGATAAAAGGTCAGCCGGGCTTGCCCGACTTTAGGTGAATCATGATAACTCCACGAATCG
Otu0065	GTCATATGCTTGTCTCAAAGATTAAGCCATGCATGTCTAAGTATAAACTGCTTTATACTGTGAAACTGCGAATGGCTCATTAAATCAGTTATAGTTTATTTGATGGTACCTACTACTCGGATACCCGTAGTAAATCTAGAGCTAATACGTGCGTAAATCCCGACTTCTGGAAGGGACGTATTTATTAGATAAAAGGCCGACCGGGCTCTGCCCGACTCGCGGTGAATCATAATAACTTCACGAATCG
Otu0018	GTCATATGCTTGTCTCAAAGATTAAGCCATGCATGTCTAAGTATAAACTGCTTTATACTGTGAAACTGCGAATGGCTCATTAAATCAGTTATAGTTTATTTGATGGTACCTTACTACTCGGATAACCGTAGTAATTCTAGAGCTAATACGTGCGCAAATCCCGACTTCCGGAAGGGACGTATTTATTAGATAAAAGGCCGACCGGGCTTGCCCGACTCGCGGTGAATCATGATAACTTCACGAATCG
Otu0021	GTCATATGCTTGTCTCAAAGATTAAGCCATGCATGTCTAAGTATAAACTGCTTATACGGTGAAACTGCGAATGGCTCATTAAATCAGTTAGAGTTTATTTGATGGTACCTTGCTACTCGGATAACCGTAGTAAAACTAGAGCTAATACGTGCGTAAATCCCGACTCACGAAGGGACGTATTTATTAGATCCAAGACCGACCGTGCTTGCACGCTCTTGGTGAATCATGATAACTTCACGAATCG
Otu0041	GTCATATGCTTGTCTCAAAGATTAAGCCATGCATGTCTAAGTATAAACTGCTTATACGGTGAAACTGCGAATGGCTCATTAAATCAGTTAGAGTTTATTTGATGGTACCTTGCTACTCGGATAACCGTAGTAAAACTAGAGCTAATACGTGCGTAAATCCCGACTCACGAAGGGACTATTTATTAGATCCAAGACCGACCGTGCTTGCACGCTCTTGGTGAATCATGATAACTTCACGAATCG
Otu0083	GTCATATGCTTGTCTCAAAGATTAAGCCATGCATGTCTAAGTATAAACAAATTTGTACTGTAAAACTGCGAATGGCTCATTAAATCAGTTATAGTTTATTTGATAGTGACTTACTACTTGGATAACCGTGGTAATTCTGCAGCTAATACATGCGTTAAACCCCGACTTCTGGAAGGGGCGTACTTATTAGATTTAAGCCAACCCGGGCAACCGGTTTATGGTTGATTCATGATAATTTTTCGAATCG
Otu0072	GTCATATGCTTGTCTAAAGGACTAAGCCATGCATGTCTAAGTATAAGCAATTATACTTGTGAAACTGCGAATGGCTCATTAAATCAGTTATAGTTTACTTGATATTTTCTTGCTACATGGATACCGGTGGCAAAGTACCGCTAATACATGCAAACGATCCCGCAAGGGATGTATTTATTAGATACGCAAACCAACCCGGCTAGCCGGAGTATGCTGAATCATAATAACTTCACGAATCG
Otu0013	GTCATATGCTTGTCTCAAAGATTAAGCCATGCATGTCTAAGTATAAGCATTTATACTGTGAAACTGCGAATGGCTCATTAAATCAGTTATAGTTTATTTGATAGTACCTTACTACTTGGATATCCGTGGTAATTCTAGGGCTAATACATGCTAAAATAGGCGACTTCTGGAAGCCTAGTATTTATTAGATAAAAAAACCAACCCGGGCAACCGGTTTACTGGTGAATCATGATAACTTTTCGAATCG
Otu0033	GTCATATGCTTGTCTCAAAGATTAAGCCATGCATGTCTAAGTATAAACAATTCTATACTGTGAAACTGCGAATGGCTCATTAAATCAGTTATAATTTATTTGATAGTACCTTACTACAAGGATAACCGTGGTAATTCTAGAGCTAATACTTGCATAAAAGCCCGACTTCGGAAGGGTTGTATTTATTAGATAAAAAACCAACCTGGGCAACCAGTTTCTTGGTGATTCATAATAACTTCTCGAATCG
Otu0069	GTCATATGCTTGTCTCAAAGACTAAGCCATGCATGTCTAAGTATAAACACCTTTATACTGCGAAACTGCGAACGGCTCATTATATCAGTTATAATCTACTTGATCGTATCCTCTTACTTGGATAACCGTAGTAATTCTAGAGCTAATACATGCAAAAGATCCCGACTGGTGACGGAAGGGATGTATTTATTAGGTCCAAAATCAATGCTGGCCATTCGGTCAGGAAAGTTGATGATTCATAATAACT
Otu0084	GTCATATGCTTGTCTCAAAGATTAAGCCATGCAAGTCTAAGTATAAGCACTTATACTGTTAAACTGCGGAAAGCTCATTATATCAGTTATAGTTTATTTGATGGTAAACCCACTACATGGATAACCGTAGTAATTCTAGAGCTAATACATGCCTTGAAGCGGTAACTTCGGAAGCCGTGGACTTATTAGATCAAGAGCTGACCCTTTTCGGAGGAACAAGCTGAATCATAGTAATTTCGCTAATCG
Otu0055	GCATACGCTTGTCTCAAAGATTAAGCCATGCATGTCTAAGTATAAACAAATCTATACTGTGAAACTGCGAATGGCTCATTATATCAGTTATAGTTTATTTGATGGTCCTTGCTACTTGGATAACCGTAGTAATTCTAGAGCTAATACATGCATCAAGCCCCGACTTCTGGAAGGGGTGTATTTATTAGATGGAAACCAATGCGGGGCAACCCGGAATCTGGTGATTCATAATAACTTTCGGATCG
Otu0054	GCATACGCTTGTCTCAAAGATTAAGCCATGCATGTCTAAGTATAAACAAATCTATACTGTGAAACTGCGAATGGCTCATTATATCAGTTATAGTTTATTTGATGGTCCTTGCTACTTGGATAACCGTAGTAATTCTAGAGCTAATACATGCATCAAGCCCCGACTTCTGGAAGGGGTGTATTTATTAGATGGAAACCATGCGGGGCAACCCGGATCTGGTGATTCATAATAACTTTCGGATCG
